# Funding and infrastructure among large-scale clinical trials examining cardiovascular diseases in Japan: evidence from a questionnaire survey

**DOI:** 10.1186/1471-2288-11-148

**Published:** 2011-11-01

**Authors:** Hiroshi Sawata, Kiichiro Tsutani

**Affiliations:** 1 Department of Drug Policy and Management, Graduate School of Pharmaceutical Sciences, The University of Tokyo, 7-3-1, Hongo, Bunkyo-ku, Tokyo, Japan

## Abstract

**Background:**

Large-scale clinical trials with thousands of participants are often needed to evaluate the risk reductions of cardiac events and/or death. Many recent clinical trials have evaluated the incidences of cardiac events using hard endpoints, especially in cardiovascular and metabolic medicine. A high investigation cost is involved in conducting a large-scale clinical trial, and obtaining sufficient funding is essential. The infrastructural environment of clinical trials is currently inadequate in Japan. We conducted a questionnaire-based survey to address this issue. The present study sought to clarify the current situation surrounding large-scale clinical trials in terms of funding and infrastructure, and to inform discussion about improving the financial and infrastructural situation for clinical trials.

**Methods:**

We sent questionnaires to 119 sponsors of large-scale clinical trials between August 2007 and December 2007, and between July 2009 and August 2009. Answers to each question were summarized and data were statistically analyzed.

**Results:**

We received responses from the sponsors of 63 (52.9%) out of 119 trials to which questionnaires were sent. The results revealed that 25 trials (39.7%) were funded by foundations, and 21 trials (33.3%) were funded by public agencies. All of the foundations involved in conducting clinical trials, where funding sources were specified, were funded by private organizations such as pharmaceutical companies. All of the clinical trials with a cost of JPY 300 million (USD 3.27 million) or more were funded by private organizations, and none were funded solely by public agencies. The sponsors of 23 trials (36.5%) responded that the trial was 'not registered' to clinical trial registry.

**Conclusions:**

The questionnaire responses revealed that there were still many trials whose funding sources were unclear and many sponsors were unaware of their responsibilities in managing and/or financing the costs of clinical trials. These findings indicate that further discussion is required to establish appropriate frameworks and/or rules regarding funding, while considering conflicts of interest. This discussion should take place as soon as possible to facilitate appropriate clinical trials.

## Background

In the 1970s, researchers began to conduct large-scale clinical trials in Western countries, with the incidence of cardiovascular events as endpoints [1,2]. Although large-scale clinical trials in Japan began approximately 10 years later than in Western countries, the number of large-scale clinical trials has since increased. Recently, a large number of clinical trials seeking to evaluate the incidence of cardiac events and/or death using hard endpoints have been conducted in cardiovascular and metabolic medicine in Japan.

The Japanese Acute Myocardial Infarction Prospective (JAMP) study [3] was the first non-pharmaceutical company-funded multicenter trial of a medication in Japan. The JAMP study group concluded that there was no significant improvement in outcomes associated with angiotensin-converting enzyme inhibitor administration in patients who had survived acute myocardial infarction in a Japanese study population.

After the JAMP study, we found a number of issues surrounding large-scale clinical trials [4]. In particular, the funding sources and infrastructure of clinical trials were found to be major issues. Our results indicated that financial and infrastructural resources must be maintained to adequately conduct clinical trials. However, a substantial cost is involved, and obtaining funding is thus essential for clinical trials. The infrastructural environment surrounding clinical trials is currently inadequate, although this situation is improving. At present, Japanese clinical trials are funded by various sources, including public agencies, private companies, foundations, etc. Several studies have reported that industry-funded trials potentially suffer from biased results, interpretations and conclusions [5-11]. Ridker, et al. found that cardiovascular trials reported between 2000 and 2005 that were funded by for-profit organizations were more likely to report positive findings than those funded by not-for-profit organizations [5]. Considering the current situation surrounding the medical and pharmaceutical industries, as well as the situation for researchers, it is unlikely that researchers can completely avoid conflicts of interest. As such, the International Committee of Medical Journal Editors (ICMJE) requested in 2004 that sponsors disclose information regarding trial management, including funding sources [12]. However, no comprehensive regulations are currently in place to manage conflicts of interest in Japan.

In the current study, we conducted a survey among trial sponsors, using questionnaires to elucidate the current funding and infrastructural environment surrounding large-scale clinical trials investigating cardiovascular diseases in Japan. In this article, we report the results of our survey regarding relevant issues surrounding large-scale clinical trials for cardiovascular diseases in Japan.

## Methods

We sent questionnaires to the sponsors of large-scale clinical trials. A 'sponsor' was defined as 'an individual, company, institution or organization that takes responsibility for the initiation, management and/or financing of a clinical trial' [13-15]. We targeted our questionnaires to the chairperson, or, when the sponsor was an institution or organization, to the individual with the most responsibility according to the official disclosure of the organization.

Between August 2007 and December 2007 we sent questionnaires to 90 large-scale clinical trial programs that commenced before February 2007. One-hundred and seventeen trials were found by 1) PubMed, 2) Ichushi (Japana Centra Revuo Medicina, URL: http://login.jamas.or.jp/), 3) websites of related medical societies, 4) University Hospital Medical Information Network (UMIN) Clinical Trials Registry (URL: http://www.umin.ac.jp/ctr/index-j.htm), and 5) clinicaltrials.gov (URL: http://www.clinicaltrials.gov/) on 25 February, 2007. Of 117 trials, we found addresses of sponsors for 90 trials, and we sent questionnaires to all sponsors whose addresses could be identified. In addition, 35 more trials were found using the same data sources as mentioned above on 25 July, 2009. We found addresses for sponsors of 29 of these 35 trials, and, sent questionnaires to all sponsors whose addresses could be identified between July 2009 and August 2009.

We defined 'large-scale clinical trials' as trials where the number of participants was 300 or more. If a trial was ongoing beyond the cut-off date and its planned number of participants was 300 or more, this trial was also regarded as a 'large-scale clinical trial'. We sought to include all large-scale clinical trials that examined cardiovascular and metabolism disease (i.e. cardiovascular, cerebrovascular events, and/or death) and used true endpoints as their primary endpoints. A true endpoint was an endpoint involving cardiovascular events, such as myocardial infarction, chronic heart failure, ischemic heart attack, and/or death. We sent questionnaires to the sponsors of all clinical trials that met the above criteria, except for sponsors that we did not have access to.

Our questionnaires consisted of categorical choices (Additional file 1 Figure S1). Sponsors were asked to return the questionnaires within 2 weeks, but all responses were included in our analyses, regardless of when they were returned. We analyzed all responses after checking the accuracy and appropriateness of the data, without specifying the trial or each sponsor.

We presented mean, standard deviation, median and IQR for the continuous variables, absolute numbers and percentages for binary and categorical variables. The statistical tests were used for the comparisons between the groups by funding source. P-values were calculated using Kruskal-Wallis test, and p < 0.05 was considered to be statistically significant. Data were statistically analyzed using software (R version 2.13.1).

We did not obtain an ethical approval for this study, because our study was not a medical research involving human subjects to understand the causes, development and effects of diseases and improve preventive, diagnostic and therapeutic interventions.

## Results

### Numbers of participants in large-scale clinical trials to which questionnaires were sent and those that answered

We sent questionnaires to sponsors of 119 large-scale clinical trials. We received responses from 63 trials (52.9%). Of the 90 trials to which questionnaires were sent in August 2007 - December 2007, we received responses from 53 (58.9%). Of the 29 trials to which questionnaires were sent in July 2009 - August 2009, we received responses from 10 (34.5%).

The number of participants is summarized in Table 1. Twenty-four of the 63 responding trials (38.1%) involved less than 1,000 participants, 19 (30.2%) involved 1,000 - 2,999 participants, 15 (23.8%) involved 3,000 - 9,999 participants, and three (4.8%) involved 10,000 participants or more. The results did not reveal clear differences in the numbers of participants between trials that responded relative to all target trials.

**Table 1:** Numbers of participants involved in large-scale clinical trials to which questionnaires were sent, and number of participants involved in trials that responded

Number of participants in clinical trial	Responded trial* n (%)	Not-responded trial* n (%)	**Total **^†^ n (%)
< 999	24 (38.1%)	28 (50.0%)	52 (43.7%)
1,000 - 2,999	19 (30.2%)	14 (25.0%)	33 (27.7%)
3,000 - 9,999	15 (23.8%)	7 (12.5%)	22 (18.5%)
≥ 10,000 -	3 (4.8%)	6 (10.7%)	9 (7.6%)
Unknown or no target number	2 (3.2%)	1 (1.8%)	3 (2.5%)

Total	63 (100%)	56 (100%)	119 (100%)

* Proportions (%) in all responding trials (63 trials) or all not-responded trials (56 trials) are shown.

† Proportions (%) in all trials (119 trials) are shown.

### Numbers of trials and participants by funding source

We calculated the numbers of large-scale clinical trials per funding source.

Twenty-five trials (39.7%) were funded by foundations, 21 trials (33.3%) were funded by public agencies such as the Ministry of Health, Labour, and Welfare (MHLW), 13 trials (20.6%) were funded by private organizations such as pharmaceutical companies, and 10 trials (15.9%) were self-funded. Sponsors of two trials (3.2%) reported that their trials were funded by "others", and the sponsor of one trial (1.6%) reported that the funding source was "unknown or unspecified". For this question, the sum of all frequencies did not equal 63 (100%), because multiple answers were allowed. Of the 25 trials that were funded by foundations, all trials except for those where funding was unknown or unspecified were found to be funded by private organizations (such as pharmaceutical companies) through foundations.

The number of participants by funding source is summarized in Table 2. In total, the mean number of participants was 2,582. The mean number of participants in private funding and combined funding trials were 3,202 and 3,361, respectively. In publicly funded trials the mean number was 1,348.

**Table 2:** Results of questionnaire survey

Questionnaire	Parameter	Funding source *					**p-value**^**†**^
			
		Public N = 14	Private N = 29	Combined N = 9	Others N = 11	Total N = 63	
Number of participants	N	12	29	9	11	61	0.6296
	Mean	1260.6	3301.9	3361.1	1489.4	2582.2	
	SD	991.9	4311.4	6295.9	1202.5	3910.3	
	Minimum	861.5	1748	1200	1090	1200	
	Q1	575	500	800	500	500	
	Median	2000	4418	2000	2046.5	3000	
	Q3	300	110	300	300	110	
	Maximum	3000	20000	20000	4000	20000	

Total investigation cost	JPY < 10 million(USD < 0.11 million)	0	2 (6.9%)	0	6 (54.5%)	8 (12.7%)	0.0003
	JPY 10 - 30 million (USD 0.11 - 0.33 million)	2 (14.3%)	1 (3.4%)	1 (11.1%)	1 (9.1%)	5 (7.9%)	
	JPY 30 - 100 million (USD 0.33 - 1.09 million)	4 (28.6%)	3 (10.3%)	1 (11.1%)	2 (18.2%)	10 (15.9%)	
	JPY 100 - 300 million (USD 1.09 - 3.27 million)	7 (50.0%)	10 (34.5%)	1 (11.1%)	1 (9.1%)	19 (30.2%)	
	JPY 300 million - 1 billion (USD 3.27 - 10.9 million)	0	4 (13.8%)	6 (54.5%)	0	10 (15.9%)	
	JPY 1 - 3 billion (USD 10.9 - 32.7 million)	0	2 (6.9%)	0	0	2 (3.2%)	
	JPY ≥ 3 billion (USD ≥ 32.7 million)	0	1 (3.4%)	0	0	1 (1.6%)	
	Unknown or unspecified	1 (7.1%)	6 (20.7%)	0	1 (9.1%)	8 (12.7%)	

Organization responsible for monitoring^‡^	Pharmaceutical company	0	6 (20.7%)	1 (11.1%)	1 (9.1%)	8 (12.7%)	-
	CRO (Contract research organization)	0	5 (17.2%)	2 (22.2%)	0	7 (11.1%)	
	Academic organization	5 (35.7%)	9 (31.0%)	3 (33.3%)	9 (81.8%)	26 (41.3%)	
	Others	10 (71.4%)	13 (44.8%)	6 (54.5%)	2 (18.2%)	31 (49.3%)	

Organization responsible for data management^‡^	Pharmaceutical company	0	6 (20.7%)	1 (11.1%)	0	7 (11.1%)	-
	CRO (Contract research organization)	1 (7.1%)	8 (27.6%)	0	0	9 (14.3%)	
	Academic organization	5 (35.7%)	9 (31.0%)	4 (44.4%)	8 (72.7%)	26 (41.3%)	
	Others	10 (71.4%)	10 (34.5%)	6 (54.5%)	2 (18.2%)	28 (44.4%)	

External efficacy evaluation committee	Yes	9 (64.3%)	17 (58.6%)	8 (88.9%)	6 (54.5%)	40 (63.5%)	0.5323
	No	5 (35.7%)	7 (24.1%)	1 (11.1%)	4 (36.4%)	17 (27.0%)	
	Unknown or unspecified	0	5 (17.2%)	0	1 (9.1%)	6 (9.5%)	
External safety monitoring committee	Yes	11 (78.6%)	16 (55.2%)	7 (77.8%)	6 (54.5%)	40 (63.5%)	0.6671
	No	3 (21.4%)	9 (31.0%)	2 (22.2%)	4 (36.4%)	18 (28.6%)	
	Unknown or unspecified	0	4 (13.8%)	0	1 (9.1%)	5 (7.9%)	

Biostatistician	Yes	11 (78.6%)	21 (72.4%)	7 (77.8%)	6 (54.5%)	45 (71.4%)	0.7010
	No	3 (21.4%)	6 (20.7%)	2 (22.2%)	4 (36.4%)	15 (23.8%)	
	Unknown or unspecified	0	2 (6.9%)	0	1 (9.1%)	3 (4.8%)	

Approval of IRB	Yes	14 (100%)	26 (89.7%)	9 (100%)	9 (81.8%)	58 (92.1%)	0.4785
	No	0	3 (10.9%)	0	1 (9.1%)	4 (6.3%)	
	Unknown or unspecified	0	0	0	1 (9.1%)	1 (1.6%)	

Site of registration^‡^	University hospital Medical Information Network (UMIN)	7 (50.0%)	8 (27.6%)	5 (55.6%)	4 (36.4%)	24 (38.1%)	-
	clinicaltrials.gov	7 (50.0%)	7 (24.1%)	4 (44.4%)	3 (27.3%)	21 (33.3%)	
	Japan Pharmaceutical Information Center (JAPIC)	0	1 (3.4%)	0	0	1 (1.6%)	
	Japan Medical Association	0	0	0	0	0	
	Others	1 (7.1%)	2 (6.9%)	0	0	3 (4.8%)	
	Not registered	3 (21.4%)	13 (44.8%)	3 (33.3%)	4 (36.4%)	23 (36.5%)	
	Unknown or unspecified	1 (7.1%)	0	0	1 (9.1%)	2 (3.2%)	

* Funding sources were categorized based on the answers to questionnaire regarding funding sources;

- Public: Public agency

- Private: Foundation, private organization

- Combined: More than one funding source

- Others: Self-funding, other

^† ^P-values were calculated using Kruskal-Wallis test.

^‡ ^Multiple answers were allowed. Therefore, the sum of all frequencies is not equal to 63 (100%).

The number of large-scale clinical trials by total cost is shown in Table 2. Nineteen trials (30.2%) where the cost or estimated cost was JPY 100 - 300 million (i.e. USD 1.09 - 3.27 million, calculated based on the foreign exchange rate on 20 February, 2010). Most of the trials were categorized in this range. The second- and third-most common cost categories were JPY 30 - 100 million (USD 0.33 - 1.09 million) and JPY 300 million - 1 billion (USD 3.27 - 10.9 million), respectively, with 10 trials (15.9%) fitting into each of these categories. 58.1% of the trials (32 out of 55 answered trials) cost JPY 100 million (USD 1.09 million) or more.

Regarding the relationship between funding sources and the cost of the trials, none of the clinical trials with a cost of JPY 300 million (USD 3.27 million) or more were funded solely by public agencies. The total trial costs were significantly different between trials involving different types of funding sources (p = 0.0003).

Eight trials (12.7%) responded that the source was unknown or left the question unanswered.

In an additional question, we asked details of the cost to the sponsors. The sponsors of 29 trials responded. Of these, 18 trials were funded by private sources. Nine of the 29 sponsors answered this question. Of these 9 unanswered trials, 8 trials were funded by private sources. The major reasons given for not answering were that the details were 'unknown' because some of the responders were not involved in cost management for their trials.

### Party responsible for monitoring and data management activities, and involvement of third-party

Regarding the infrastructure required for conducting large-scale clinical trials, we investigated the situations surrounding the support of human resources and material resources in trials whose sponsors reported foundations or private organizations as funding sources. Of 63 trials, 37 trials were private-funded. Nineteen of 37 trials (51.4%) received human or material resources from other organizations. Sixteen trials (43.2%) were supported with human resources only, one trial (2.7%) was supported with material resources only, and one trial (2.7%) was supported with both human and material resources. Fifteen trials (37.0%) were not supported with human or materials resources, receiving only financial resources. Three trials (8.1%) either reported that their support was unknown or left the question unanswered.

The number of large-scale clinical trials sorted by the party responsible for monitoring and data management activities is shown in Table 2. Twenty-six trials (41.3%) were conducted by academic organizations, while 'others' (such as trial secretariats) were reported most frequently, with 31 and (49.3%) 28 trials (44.4%) reporting monitoring and data management activities, respectively. Monitoring in eight trials (12.7%) and data management in seven trials (11.1%) were conducted by pharmaceutical companies, and monitoring of seven trials (11.1%) and data management of nine trials (14.3%) were conducted by a contract research organization (CRO).

Some sponsors commented that a lack of available time of external and internal staff at the trial sites was one of the major reasons for delays in subject enrolment.

The numbers of large-scale clinical trials that involved external committees, biostatisticians and institutional review boards (IRBs) are shown in Table 2. Forty trials (63.5%) were associated with external committees to maintain objectivity in efficacy evaluations. Forty trials (63.5%) were associated with external committees to monitor the safety of the drugs or treatments and/or to avoid harmful outcomes for subjects. Forty-five trials (71.4%) involved biostatisticians. Fifty-eight trials (92.1%) were approved by IRBs.

### Clinical trial registry

The number of large-scale clinical trials by site of clinical trial registry is shown in Table 2. The UMIN and clinicaltrials.gov were the major sites of registration, used for the registration of 24 trials (38.1%) and 21 (33.3%) trials, respectively. Only one trial (1.6%) was registered by the Japan Pharmaceutical Information Center (JAPIC) and none by the Japan Medical Association. The sponsors of 23 trials (36.5%) responded that the trial was 'not registered'.

## Discussion

Our examination of the funding of large-scale clinical trials in cardiovascular and metabolic diseases in Japan revealed that sponsors of large-scale clinical trials in Japan received funding from various sources but some of them were unaware of their responsibilities in managing and/or financing the costs of clinical trials. Of the trials funded by foundations, all trials except for those reporting that their funding source was unknown or unspecified were funded by private organizations such as pharmaceutical companies, through these foundations. This finding indicates that there are still many trials whose funding sources are unclear. Regarding the infrastructure for operating large-scale clinical trials, more than half of the trials received support in the form of human and/or material resources in trials funded by foundations/private organizations. Most of the support from foundations/private organizations was in the form of human resources. Some sponsors commented that a lack of available time of external and internal staff at the trial sites was one of the major reasons for delays in subject enrolment. These results suggest that the current situation should be improved by providing sufficient human resources for clinical trials. In about one third of the trial, third parties, such as independent efficacy/safety evaluation committees and biostatisticians, were not involved. Thus, there is room for improvement, although these frequencies are relatively high. One of the limitations of our study was that some of the large-scale clinical trials in Japan might have not been included due to lack of publications through clinical trial registries.

Sample sizes were similar to those reported in a review of clinical trials in Western countries between 2000 and 2005 [5], where the mean and median numbers of participants were 560-4,239 and 421-1,486, respectively, although the number of participants was found to vary between trials with different funding sources.

Approximately one third of the responding trials were registered by the UMIN and clinicaltrials.gov, respectively. These sites for registration were both recommended for clinical trial registry by the International Committee of Medical Journal Editors (ICMJE) [16]. Our findings suggest that some sponsors are not aware of the importance of clinical trial registration. ICMJE in 2004 [12], the Declaration of Helsinki revised in 2008 [17] and the CONSORT declaration in 2010 [18] request the disclosure of trial protocol summaries and results by sponsors of clinical trials to avoid publication biases. Considering the purpose of clinical trial registration, we wish to emphasize that sponsors should be aware an important responsibility of sponsors is to minimize publication bias, thus improving the quality of evidence produced by large-scale clinical trials.

Evidence from clinical trials using true endpoints such as cardiovascular events is important, despite the high costs involved. We found that the currently available evidence was obtained in trials receiving funding from various sources, and requiring various infrastructural resources. However, the potential effects of funding sources on the results of clinical trials represent a major problem, potentially contributing to lower reliability of clinical practice guidelines. Previous trials in Western countries reported that industry-supported meta-analyses were less transparent than meta-analyses with non-profit support or no support [5-11]. In 2008, the Association of American Medical Colleges reported an examination of the relationship between academic institutions and pharmaceutical industries, and recommended that discussions should be undertaken by committees consisting of both academics and pharmaceutical industry staff to improve the transparency of clinical trials [19]. The transparency of clinical trials is important for minimizing potential bias in results, particularly in trials funded by pharmaceutical companies. As a first step, we wish to highlight the importance of revealing funding and infrastructural sources, as well as the importance of clinical trial registration by sponsors and their supporters. In addition, ways of improving education related to financial bias and publication bias in medical programs at universities and medical colleges should be seriously considered.

One limitation of the current study was that we did not receive responses from the sponsors of approximately 40% of the trials we initially identified. In addition, it was not clarified where the money was allocated in each trial. Therefore, this survey did not allow for conclusions on whether expensive trials requested the additional amount of support to recruit more staff, use more sophisticated methods and high technology resources, or they had just to pay extra money for honorariums and travel expenses, which probably do not contribute much to the improvement of the validity of clinical trials.

## Conclusions

The results of the current study revealed that there were still many trials whose funding sources were unclear and many sponsors were unaware of their responsibilities in managing and/or financing the costs of clinical trials. As such, we propose the establishment of rules regarding funding and human resource-related support structures, e.g. requirements for concurrent funding from multiple sources, clarification of legal requirements, requirements for thorough disclosure of conflicts of interest, promotion of internal human resource use and outsourcing, and clarification of the responsibilities of individuals involved in conducting trials. These results can contribute to the improvement of conflict-of-interest management in Japan as well as other countries. This discussion should take place as soon as possible to facilitate appropriate clinical trials. After such an improvement, we will be able to investigate more efficient allocation of various resources to improve the validity of clinical trials and quality of evidences from clinical trials.

## Competing interests

HS is an employee of Novartis Pharma K.K. The Department of Drug Policy and Management, Graduate School of Pharmaceutical Sciences, The University of Tokyo, is a department endowed by Towa Pharmaceutical Co., Ltd., one of the leading manufacturers of generic drugs in Japan.

## Authors' contributions

HS examined the study and drafted the manuscript. KT helped to draft the manuscript. All authors read and approved the final manuscript.

## Pre-publication history

The pre-publication history for this paper can be accessed here:

http://www.biomedcentral.com/1471-2288/11/148/prepub

## Supplementary Material

Additional file 1 **Answer Sheets for Questionnaire Survey Regarding Executions of Large-Scale Clinical Trials Evaluating Cardiovascular Events as Endpoint**. These answer sheets are the English translated versions used in our survey. The answer sheets distributed to sponsors were written in Japanese. Click here for file
