# Supplementary material for: Funding and infrastructure among large-scale clinical trials examining cardiovascular diseases in Japan: evidence from a questionnaire survey
Source: BMC Med Res Methodol. 2011 Nov 1;11:148. doi: 10.1186/1471-2288-11-148 (PMC3228708; doi:10.1186/1471-2288-11-148)
Supplement: Additional file 1 — Answer Sheets for Questionnaire Survey Regarding Executions of Large-Scale Clinical Trials Evaluating Cardiovascular Events as Endpoint. These answer sheets are the English translated versions used in our survey. The answer sheets distributed to sponsors were written in Japanese. [file 1471-2288-11-148-S1.DOC]

## Figure S1 – Answer sheets for questionnaire survey regarding executions of large-scale clinical trials evaluating cardiovascular events as endpoint

Questionnaire Survey Regarding Executions of Large-Scale Clinical Trials Evaluating Cardiovascular Events as Endpoint

Answer Sheet A

Final ver. 1.0 (2009.7.23)

Please use **one set of Answer Sheet A per person**. Please fill a section with “−” when the answer is unknown or unable to answer.

Q1: Please confirm an official title and an abbreviated title of the large-scale clinical trial(s) which you are conducting (or conducted). If you are conducting (or conducted) other large-scale clinical trial(s) as ‘sponsor’ ^*1)^’ which are not listed here but in scope ^*2)^ of our survey, please fill the title(s) of trial(s).

| *1) | In general, funding organizations are called ‘sponsors.’ However, in this study, they are not funding organizations of the trial costs but the individuals or groups which take responsibility for initiating, managing and/or financing trials. Please be careful about that.  WHO defines the International Clinical Trial Registry Platform based on the WHO-GCP. In it, ‘Primary Sponsor’ of the clinical trial is defines as follows (http://www.who.int/ictrp/data_set/en/index.html).  Primary sponsor: The individual, organization, group or other legal entity which takes responsibility for initiating, managing and/or financing a study. The Primary Sponsor is responsible for ensuring that the trial is properly registered. The Primary Sponsor may or may not be the main funder.  UMIN-CTR, which is one of the major clinical trial registry (CTR) systems in Japan, ‘Sponsor’ is defined as follows (http://www.umin.ac.jp/ctr/UMIN-CTR_Yougo.htm).  Sponsor: Organization which takes responsibility for managing a study including planning a study, analyzing, publishing results, and arranging study costs. |
| --- | --- |
| *2) | Large-scale clinical trials in scope of our survey: trials where the numbers of participants are 300 or more, and where primary endpoints are cardiovascular events. |

[Large-scale clinical trial(s) in scope of our survey]

1. XXX
2. YYY

If you are conducting (or conducted) other trial(s), please fill below.

　(3) 　　　　　　　　　　　　　　　　　　(Abbreviated title, if applicable: 　　　　　　　　　)

　(4) 　　　　　　　　　　　　　　　　　　(Abbreviated title, if applicable: 　　　　　　　　　)

　(5) 　　　　　　　　　　　　　　　　　　(Abbreviated title, if applicable: 　　　　　　　　　)
If you have further additions, please list on an additional sheet (same for the following questions).

Q2: Who prepared the designs of trials listed in Q1? Or what organization prepared the designs of trials?

　(1)

　(2)

　(3)

　(4)

　(5)

Q3: Please fill your contact in case we confirm something or we ask questions regarding your answers.

　Name who answered: 　　　　　　　　　　　　Affiliation:

　tel: 　　　　fax: 　　e-mail:

Thank you. Please go to Answer Sheet B.

Questionnaire Survey Regarding Executions of Large-Scale Clinical Trials Evaluating Cardiovascular Events as Endpoint

Answer Sheet B

Final ver. 1.0 (2009.7.23)

Please use **one set of Answer Sheet B per trial**. Please fill a section with “−” when the answer is unknown or unable to answer.

Trial title: XXX

Q1: What organization(s) directly funded the sponsor to conduct this trial? (Multiple answers are allowed.) Please mark ‘’ on the number. If multiple organizations funded the sponsor, please answer funded periods by organization. It does not matter which procedures, i.e. contract, subsidization, donation, etc., were applied.

| *Sponsor | ‘Sponsor’ is defined as individuals or organizations which takes responsibility for initiating, managing and/or financing a study, as shown in Answer Sheet A. |
| --- | --- |

(1)　Public agency (Ministry of Health, Labour and Welfare ; Ministry of Education, Culture, Sports, Science and Technology ; Ministry of Agriculture, Forestry and Fisheries ; Ministry of Economy ; Others (Mark ‘○’ on either of them)

(2) Foundation (3) Scientific society (4) NPO (5) Private organization (profiting company, etc.)

(6) Self-funding (7) Others →(Please specify)

Q2: **In the case that answers of Q1 were ’(2) Foundation’, ‘(3) Scientific society’ or ‘(4) NPO’ only,** what organization(s) funded to the funding organizations for this trial, if other organizations funded to the organizations answered in Q1? (Multiple answers are allowed.) Please mark ‘○’ on the number.

(1)　Public agency (Ministry of Health, Labour and Welfare ; Ministry of Education, Culture, Sports, Science and Technology ; Ministry of Agriculture, Forestry and Fisheries ; Ministry of Economy ; Others (Mark ‘○’ on either of them)

(2) Foundation (3) Scientific society (4) NPO (5) Private organization (profit-making company, etc.)

(6) Self-funding (7) Others →(Please specify)

Q3: What is the total cost of this trial? (Please answer the actual cost spent for a completed/finished trial. Please answer the budget for a planning or ongoing trial. Please include personnel expenses, honorariums, system construction costs for patient registration and database, travel expenses, expenses for consumable goods, outsourcing costs (indirect costs, personnel expenses, etc.) as much as possible.)

　(1)　< 10 million JPY 　(2)　10 million ~ 30 million JPY

　(3)　30 million ~ 100 million JPY 　(4)　100 million ~ 300 million JPY

　(5)　300 million ~ 1 billion JPY 　(6)　1 billion ~ 3 billion JPY

　(7)　≥ 3 billion JPY

Q4: What are the details of trial costs? (Please answer the actual cost spent for a completed/finished trial. Please answer the budget for a planning or ongoing trial.) Please answer the actual costs spent or proportions to the whole trial costs by items below.

Personnel expenses 　　　　　　　　　　JPY or 　　　　　　%
(Costs for clerks, accountants, CRCs, data managers, etc.)

Honorariums 　　　　　　　　　　JPY or 　　　　　　%
(Paid honorariums to investigators)

System construction costs 　　　　　　　　　　JPY or 　　　　　　%
(System construction and management costs for registration centers and/or databases for data management)

Travel expenses 　　　　　　　　　　JPY or 　　　　　　%
(Expenses for traveling for meeting with investigators, travelling expenses of CRCs from/to medical institutions)

Expenses for consumable goods 　　　　　　　　　　JPY or 　　　　　　%
(Expenses for printing and consumable goods)

Outsourcing costs 　　　　　　　　　　JPY or 　　　　　　%
(Costs for CRC activities, secretariats, data management activities, and other outsourced activities)

Q5: **In the case that answers of Q1 were ’(2) Foundation’, ‘(3) Scientific society’, ‘(4) NPO’, or ‘(5) Private organization’ only,** were resources other than monetary ones, e.g. humans or equipments, provided? Please mark ‘○’ on the appropriate answer.

　　　( Yes, No )

Q6: **In the case that answers of Q5 was ‘Yes’ only,** what were they? (Multiple answers are allowed.) Please mark ‘○’ on the number.

　(1) Supports of human resources (2) Supports of equipments (3) Other supports

Q7: What organizations conducted monitoring (verifying and gathering the data) at medical institutions? (Multiple answers are allowed.) Please mark ‘○’ on the number.

　(1) Pharmaceutical company (2) CRO (3) Academic institution (university, etc.) (4) Others

Q8: What organizations conducted data management (statistical analyses of the data)? (Multiple answers are allowed.) Please mark ‘○’ on the number.

　(1) Pharmaceutical company (2) CRO (3) Academic institution (university, etc.) (4) Others

Q9: Did you have external committee(s) to objectify the efficacy evaluations? Please mark ‘○’ on the appropriate answer.

　　　( Yes, No )

Q10: Did you have external committee(s) to objectify the safety evaluations? Please mark ‘○’ on the appropriate answer.

　　　( Yes, No )

Q11: Were biostatisticians involved in planning and/or execution of this trial? Please mark ‘○’ on the appropriate answer.

　　　( Yes, No )

Q12: Have this trial been reviewed and approved by an IRB or an ethics committee? Please mark ‘○’ on the appropriate answer.

　　　( Yes, No )

Q13: Did you register this trial to the clinical trial registry(ies)? Please mark ‘○’ on the number. (Multiple answers are allowed.)

　(1) UMIN Clinical Trial Registry (Including retroactive registrations)
(http://www.umin.ac.jp/ctr/index-j.htm)

　(2) Japan Pharmaceutical Information Center (JAPIC)
(http://www.clinicaltrials.jp/user/cte_main.jsp)

　(3) The Japan Medical Association Center for Clinical Trials (https://dbcentre2.jmacct.med.or.jp/ctrialr/)

　(4) clinicaltrials.gov (http://clinicaltrials.gov/)

　(5) Others Please specify:

　(6) Not registered

Q14: Please specify the problems you are facing or you faced during a large-scale clinical trial, if any. (If you need more spaces, please use additional sheets.)

Q15: Please write your comments freely regarding large-scale clinical trials in Japan. (If you need more spaces, please use additional sheets.)

Questionnaires were completed. Thank you for your cooperation.
